# Moral decision-making at night and the impact of night work with blue-enriched white light or warm white light: a counterbalanced crossover study

**DOI:** 10.1080/07853890.2024.2331054

**Published:** 2024-04-18

**Authors:** Erlend Sunde, Anette Harris, Olav Kjellevold Olsen, Ståle Pallesen

**Affiliations:** a Department of Psychosocial Science, University of Bergen, Bergen, Norway; b Department of Leadership and Organizational Behaviour, BI Norwegian Business School, Bergen, Norway; c Norwegian Competence Center for Sleep Disorders, Haukeland University Hospital, Bergen, Norway

**Keywords:** Shift work, sleep deprivation, colour temperature, light emitting diode

## Abstract

**Background:**

Cognitive function, including moral decision-making abilities, can be impaired by sleep loss. Blue-enriched light interventions have been shown to ameliorate cognitive impairment during night work. This study investigated whether the quality of moral decision-making during simulated night work differed for night work in blue-enriched white light, compared to warm white light.

**Methods:**

Using a counterbalanced crossover design, three consecutive night shifts were performed in blue-enriched white light (7000 K) and warm white light (2500 K) provided by ceiling-mounted LED luminaires (photopic illuminance: ∼200 lx). At 03:30 h on the second shift (i.e. twice) and at daytime (rested), the Defining Issues Test-2, assessing the activation of cognitive schemas depicting different levels of cognitive moral development, was administered. Data from 30 (10 males, average age 23.3 ± 2.9 years) participants were analysed using linear mixed-effects models.

**Results:**

Activation of the post-conventional schema (P-score), that is, the most mature moral level, was significantly lower for night work in warm white light (EMM; estimated marginal mean = 44.3, 95% CI = 38.9–49.6; *p*^holm^=.007), but not blue-enriched white light (EMM = 47.5, 95% CI = 42.2–52.8), compared to daytime (EMM = 51.2, 95% CI = 45.9–56.5). Also, the P-score was reduced for night work overall (EMM = 45.9, 95% CI = 41.1–50.8; *p*=.008), that is, irrespective of light condition, compared to daytime. Neither activation of the maintaining norms schema (MN-score), that is, moderately developed moral level, nor activation of the personal interest schema (i.e. the lowest moral level) differed significantly between light conditions. The MN-score was however increased for night work overall (EMM = 26.8, 95% CI = 23.1–30.5; *p*=.033) compared to daytime (EMM = 23.1, 95% CI = 18.9–27.2).

**Conclusion:**

The results indicate that moral decisions during simulated night work in warm white light, but not blue-enriched white light, become less mature and principle-oriented, and more rule-based compared to daytime, hence blue-enriched white light may function as a moderator. Further studies are needed, and the findings should be tentatively considered.

*Trial registration:* ClinicalTrials.gov (ID: NCT03203538) Registered: 26/06/2017; https://clinicaltrials.gov/study/NCT03203538

## KEY MESSAGES

- The quality of moral decision-making, seen as the activation of cognitive schemas depicting different levels of moral development, was reduced during simulated night work in warm white light, but not blue-enriched light, compared to daytime.
- The quality of moral decision-making sems to be reduced during simulated night work, compared to daytime.
- More studies assessing the impact of night work and light interventions on the quality of moral decision-making are needed to validate these tentative findings.

## Introduction

Moral decision-making refers to the ability to reason, judge, and make decisions regarding moral issues or principles [1]. In some occupations, such as healthcare, police, and military, workers may face imminent ethical dilemmas that necessitate thoughtful moral decision-making. Furthermore, to sustain 24-h service, night work is common in these occupations. Night work is associated with circadian misalignment and disturbed sleep/sleep loss (e.g. acute and partial sleep deprivation), sleepiness, and impaired cognitive performance during night work [2–4]. Basic cognitive functions such as vigilant attention are impaired by sleep loss, but also higher-order cognitive processes appear to be negatively affected [5, 6]. Higher-order cognitive functions rely heavily on the prefrontal cortex (PFC), a brain region considered especially vulnerable to sleep loss [7]. The PFC develops and matures into adulthood [8] and has been linked to moral decision-making [1]. In line with this, studies have shown that activity in PFC regions increases when judging moral dilemmas [9, 10]. However, following sleep deprivation, there is a global decrease in brain activity, with larger reductions in PFC regions [11]. Still, few studies have investigated moral decision-making during sleep deprivation. The authors of one study found that 53 h of sleep deprivation slowed the responses to moral personal dilemmas (i.e. difficult and emotionally evocative), but not impersonal dilemmas [12]. However, another study did not find such effects on moral decision-making after one night of sleep deprivation [13]. In a study on partial sleep deprivation (i.e. sleep restricted to 2.5 h per night over 5 days), the quality of moral decision-making changed, as moral decisions became more rule-focused, and the ability to engage in higher-level principle-oriented reasoning was reduced [14]. It has also been shown that partial sleep deprivation reduces the ability to anticipate moral problems [15], and that lack of sleep reduces moral awareness [16]. However, no study has investigated the influence of night work on moral cognition.

Evaluation of the quality of moral decision-making is commonly based on theories of cognitive moral development (CMD). One theory, referred to as the neo-Kohlbergian approach, proposes three cognitive schemas that depict different levels of CMD [17]. The most mature moral level (i.e. the highest level of CMD) has been defined as the post-conventional schema [17]. Here, moral decision-making is based on universal ideals and principles of justice and fairness and requires higher-order cognitive processes to deduce moral principles and infer moral consequences in a situation. The schema of maintaining norms represents a moderate level of CMD, where moral decision-making is based on rules and regulations and motivated by an aim to maintain stability and established social order [17]. Representing the lowest level of CMD, the personal interest schema orients moral decision-making towards personal gains and is considered a more primitive form of moral reasoning [17]. Notably, individuals may activate different moral schemas in a specific situation. However, the most morally mature have more frequent activation of the post-conventional schema, which is relevant as there is a clear link between activation of this mature schema and moral behaviors, compared to the lower schemas [17]. In general, females have higher post-conventional schema activation than males, while maintaining norms and personal interest schema activation is higher for males than females [18].

Several measures have been proposed to ameliorate cognitive performance impairment during night work, including bright-light therapy [19]. Light interventions have regained interest with the development of cost-effective light-emitting diode (LED) technology, accompanied by advances in the understanding of how light affects humans *via* non-image forming (NIF) responses [20,21]. In terms of cognitive function and performance, light exposure can have direct effects, for example, by alerting responses [22,23], as well as indirect effects, for example, *via* circadian regulation and/or effects on sleep [24]. Furthermore, these NIF responses depend on light characteristics such as the timing of exposure, light intensity, and spectral distribution [25]. Spectral distribution is important because NIF responses are mediated by intrinsically photosensitive retinal ganglion cells (ipRGCs) expressing the photopigment melanopsin, which is most sensitive to short-wavelength (i.e. blue-appearing) light [20]. Thus, blue-enriched (i.e. short-wavelength enriched) light can reduce sleepiness and cognitive performance impairments during night work [26–30]. However, it has been noted that findings are somewhat inconsistent between laboratory and field studies [20], and most previous studies have limited their focus to effects on subjective alertness, behavioral alertness (e.g. attention tests), and endogenous circadian rhythms (e.g. melatonin rhythm). To the best of our knowledge, no previous study has assessed the quality of moral decision-making during night work and whether it is affected by light interventions.

The aim of the present study was to investigate whether the quality of moral decision-making, seen as the activation of moral schemas, differed during simulated night work in blue-enriched white light (7000 K) compared to warm white light (2500 K). It was also assessed whether the quality of moral decision-making differed during simulated night work compared to daytime.

## Methods

### Participants, study design and procedures

Participants were recruited mainly among university students that were screened prior to enrollment. All participants reported good health, normal sleep (6–10 h per night, waking up before 10:00 h), and no recent night work or transmeridian travel. Participants’ adherence to the sleep criteria (i.e. waking up before 10:00 h and 6–10 h sleep per night) was confirmed with actigraphy and sleep diaries for three days prior to the simulated night work.

**Figure 1.**
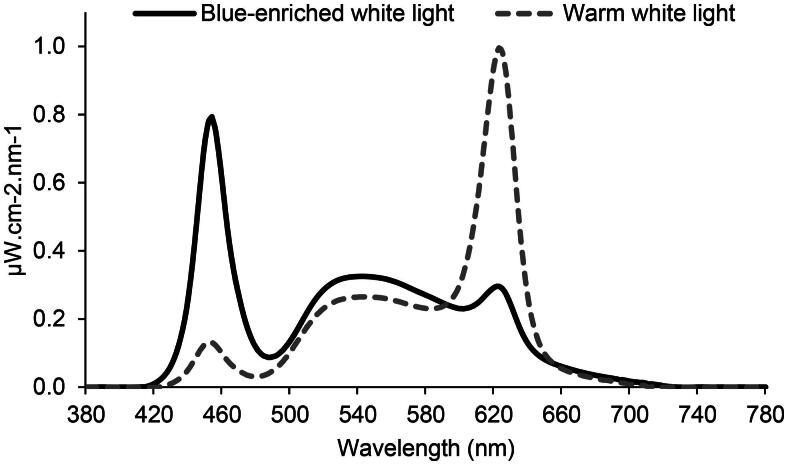
Spectral distribution of the two light conditions.

This study was conducted between January and April 2018. A counterbalanced crossover study design was employed, with participants working three consecutive night shifts (23:00–06:45 h, Friday–Monday) twice in a light laboratory (30 m^2^, no windows) at the Faculty of Psychology, University of Bergen, Norway. Participants slept at home and came to the laboratory in groups of seven to nine (four groups in total) to work the night shifts. Two groups (approximately half of the participants) started with night shifts in blue-enriched white light, while the others started with warm white light. After four weeks of washout, the participants repeated the simulated night work under the opposite light condition. The laboratory was equipped with 20 tunable ceiling-mounted LED luminaires (Modul R 600 LED CCT/RGB MP; Glamox Luxo Lighting AB, Sweden; size 60 cm x 60 cm), with two luminaires situated directly above each of the nine workplaces. Each workplace, separated by partition walls, had a desk and computer screen fitted with a physical filter blocking all light wavelengths <520 nm (Metolight SFG-10; Asmetec, Germany). Light measurements were conducted at each of the nine workplaces, approximately at eye level while seated (i.e. vertically, 120 cm above the floor) and facing the computer screen (turned on). A calibrated spectroradiometer (GL Spectics 1.0 T Flicker; GL Optic, Poland) was used for these measurements. Light measures were calculated in accordance with CIE S 026:2018 [31], using the CIE S 26 Toolbox, and the averages for the nine workplaces are presented. Thus, the reported light levels are not precise/exact exposure levels for each participant but are representative for the uniformly lit environment. The blue enriched white light (7000 K) and warm white light (2500 K) exhibited mean (SD) photopic illuminances of 197 lx (19 lx) and 206 lx (18 lx), respectively. The light conditions had similar photon densities (∼1.6 x 10^14^ photons/cm^2^/s), and the mean (SD) melanopic EDI (equivalent daylight illuminance) was 174 lx (17 lx) and 78 lx (7 lx) for blue-enriched white light and warm white light, respectively. The spectral distribution is provided in Figure 1.

During the night shift, participants were mainly seated at a designated workplace (the same workplace during each time in the laboratory) where they completed various cognitive tests and questionnaires. There were several short breaks, during which the participants could engage in quiet activities such as reading and conversation. At 03:30 h on the second night shift the Defining Issues Test-2 (DIT-2) was administered, and participants used approximately 30 min for completion (see DIT-2 details below). DIT-2 was also administered at daytime, three days after the last period with simulated night work. Thus, DIT-2 was completed three times, twice during simulated night work (i.e. in blue-enriched white light and warm white light) and once during daytime. During the three nights after the last night shift (i.e. before the daytime assessment), recovery sleep was monitored with actigraphy and sleep diary.

This study was part of a larger study registered at ClinicalTrials.gov (NCT03203538). All participants provided written informed consent and the study was approved by REC west (the Regional Committee for Medical and Health Research Ethics, health region West, Norway), application number: 2016/1903, application ID: 17308. The study follows the principles of the Declaration of Helsinki. More details of the participants, study design, light conditions, and procedures have been reported previously [28].

### The Defining Issues test-2

The DIT-2 is an instrument used to assess moral judgment by activating moral schemas and measuring schema activation [32]. A Norwegian translation of DIT-2 was used [33]. DIT-2 consists of five moral dilemmas that the participants are instructed to evaluate by rating 12 items according to their value for making a decision. A 5-point Likert scale ranging from “no importance” to “great importance” was used, and the participants were also asked to rank the four most important items for making their decision. The results of the rating and ranking of arguments are used to assess which moral level the participants are likely to use for moral decision-making. The scores for the three CMD schemas according to Rest et al. [17] are indexed in terms of percentages of the post-conventional score (P-score), maintaining norms score (MN-score), and personal interest score (PI-score), respectively [32]. DIT-2 also includes scoring of “meaningless items” (M-score), which is used to detect respondents who do not choose items based on their meanings (e.g. trying to fake a good score), and an M-score above 8 may provide grounds for exclusion [32].

### Statistical analysis

Data from 30 participants were included in the analyses, but the dataset was incomplete. Two participants’ tests/datasets from the blue-enriched white light, two from the warm white light, and one from the daytime condition were excluded due to non-compliance with the sleep criteria (*n* = 2) or high M-score (≥14, *n* = 3). In addition, daytime data from two participants were excluded because they had <6 h sleep duration the night before the daytime assessment. Also, data from one participant was missing (due to illness) from the warm white light, and two were missing (did not complete DIT-2) from the daytime condition. Thus, data from 28, 27, and 25 participants were included for the blue-enriched white light, warm white light, and daytime conditions, respectively. Twenty-five participants completed both light conditions, and 20 completed all three conditions (warm white light, blue-enriched white light, and daytime).

The data were analyzed with linear mixed-effects models (LMMs) using the lme4 and lmerTest packages in *R* [34–36]. The P-score, MN-score, and PI-score were treated as dependent variables and analyzed separately. All LMMs were fitted with random intercepts at the subject-level. The condition (warm white light vs. blue-enriched white light vs. daytime) was used as a fixed effect. To control for any effects of the order of conditions, Order (starting with warm white light vs. starting with blue-enriched white light) and the Condition × Order interaction were also added as fixed effects. Furthermore, the effects of Sex (male vs. female) and Age (centered) were examined. The final model was selected according to the best fit using likelihood ratio tests, and the final model was estimated using the restricted maximum likelihood. Model assumptions were checked by assessment of residual plots, and normality of residuals was assessed using the Shapiro-Wilk normality test. Violation of the normality of residuals assumption was indicated for the PI-score. One outlier was identified and excluded, and after re-analysis, the Shapiro-Wilk test indicated normality of residuals. Estimated Marginal Means (EMMs) with 95% confidence intervals (Cis) were calculated using the Kenward-Roger method, and multiple comparisons were adjusted using the Holm-Bonferroni method [37]. Unadjusted and adjusted *p*-values (α=.05) are reported. To assess whether there was an overall effect of night work, the two light conditions were collapsed into one night work category as a Time-of-day (night work vs. daytime) variable. The LMM procedure was repeated as described above, with the Time-of-day variable replacing the Condition variable. The model comparison and building process are reported in Supplementary Material (Tables S1–S3).

**Table 1.** Linear mixed model analyses of activation of the post-conventional schema (P-score), maintaining norms schema (MN-score), and personal interests schema (PI-score).

	P-score					MN-score					PI-score				
*Predictors*	*Estimates*	*95% CI*	*Statistic*	*df*	*p (p^holm^)*	*Estimates*	*95% CI*	*Statistic*	*df*	*p (p^holm^)*	*Estimates*	*95% CI*	*Statistic*	*df*	*p (p^holm^)*
Intercept	44.25	38.87 − 49.63	16.57	45.49	<0.001	30.63	24.11 − 37.14	9.53	36.13	<0.001	18.58	15.12 − 22.04	10.72	64.68	<0.001
**Condition**															
Warm white light (*n* = 27)	*Ref.*					*Ref.*					*Ref.*				
Blue-enriched white light (*n* = 28)	3.26	−1.11 − 7.63	1.50	49.14	0.140 (0.140)	−1.43	−5.37 − 2.50	−0.73	49.62	0.468 (0.468)	−0.01	−3.87 − 3.88	−0.00	49.73	0.996(1.000)
Daytime (*n* = 27)	7.00	2.49 − 11.51	3.12	49.07	0.003 (0.006)	−4.52	−8.58 – −0.46	−2.24	49.61	0.030 (0.119)	−0.99	−0.99 − 3.01	−0.50	49.95	0.620(1.000)
**Sex**															
Male						*Ref.*									
Female						−4.59	−12.08 − 2.90	−1.26	27.11	0.219 (0.439)					
**Age** (centered)						2.91	−0.61 − 6.43	1.70	26.27	0.102 (0.305)					
**Random Effects**															
Residual		62.17				50.62					48.27				
Subject		141.25				65.58					31.79				
ICC		0.69				0.56					0.40				
N / N observations		30 / 80				30 / 80					30 / 79				
Marginal R^2^ / Conditional R^2^		0.038 / 0.706				0.135 / 0.624					0.003 / 0.399				

Results from the final models with best fit according to likelihood ratio tests (for PI-score no fixed factors improved model fit).

*CI* confidence interval, *df* degrees of freedom*, p^holm^* Holm-Bonferroni adjusted P-value, *Ref.* reference, *ICC* intraclass correlation coefficient.

## Results

In total, 30 (10 males) young (average age = 23.3 years, SD = 2.9 years) adult participants were included. There were no significant Condition × Order interaction effects for either P-score (*p*=.424), MN-score (*p*=.206), or PI-score (*p*=.770). Thus, no significant crossover or order effects were observed. The average sleep duration for three nights prior to the first night shift (i.e. baseline) was 7:49 h (SD = 0:49 h) and 7:40 h (SD = 1:01 h) for warm white light (*n* = 27) and blue-enriched white light (*n* = 28), respectively. The average recovery sleep duration (i.e. for three nights before the daytime assessment; *n* = 25) was 7:50 h (SD = 0:59 h).

**Figure 2.**
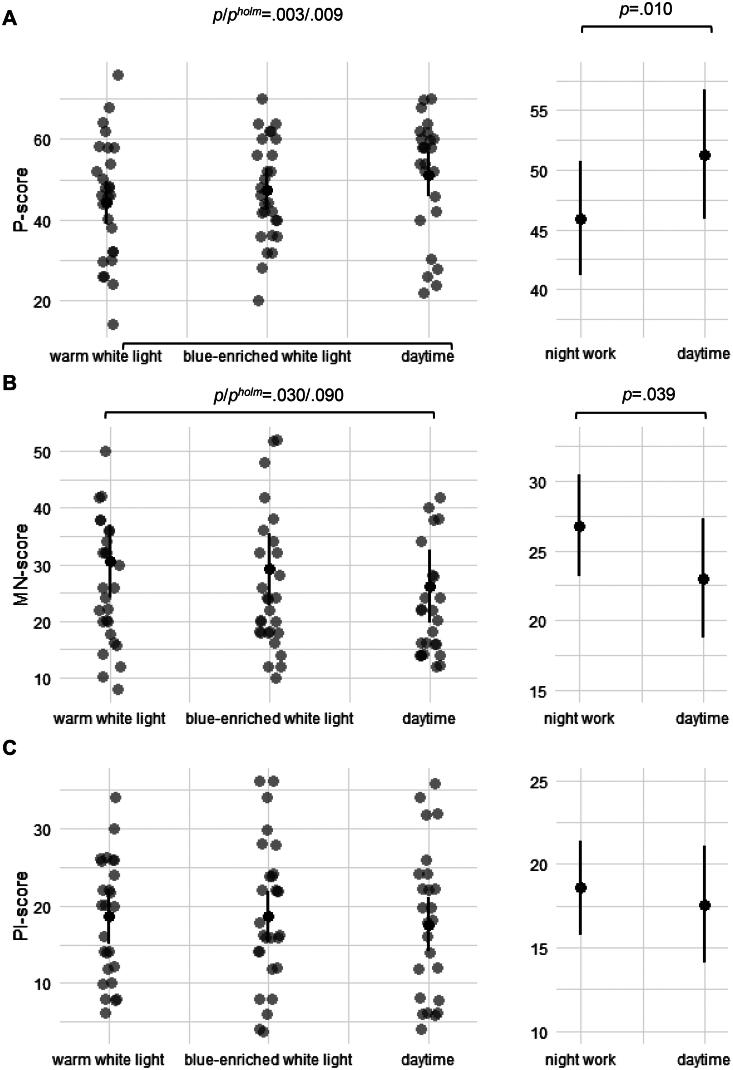
Activation of (A) post-conventional schema (P-score), (B) maintaining norms schema (MN-score) and (C) personal interests schema (PI-score). Left panels shows raw scores (grey dots) and Estimated Marginal Means (EMMs) with 95% CI (black dots and error bars) during night work (at 03:30  h) in warm white light and blue-enriched white light, and during daytime. Right panels shows EMMs with 95% CI during night work (light conditions collapsed) and daytime.

The final model for the P-score (Table 1, Figure 2(A); left panel) showed a significant effect of Condition (F_2,49.23_=4.86, *p*=.012), with a reduced P-score for night work in warm white light (EMM = 44.3, 95% CI = 38.9–49.6; *p*/*p*^holm^=.003/.009) compared to daytime (EMM = 51.2, 95% CI = 45.8–56.7). The P-score for blue-enriched white light (EMM = 47.5, 95% CI = 42.2–52.8) did not differ significantly (*p*/*p*^holm^=.102/.203) compared to daytime. The P-score during night work did not differ significantly (*p*/*p*^holm^=.140/.203) between light conditions. For the final model with Time-of-day (Figure 2(A); right panel) used as a fixed factor, there was a significant effect of Time-of-day (F_1,50.41_=7.21, *p*=.010, Marginal R^2^/Conditional R^2^=0.030/0.690), with a reduced P-score for night work (EMM = 45.9, 95% CI = 41.1–50.8) compared to daytime (EMM = 51.3, 95% CI = 45.8–56.7).

For the MN-score, the final model (Table 1, Figure 2(B); left panel) included Condition (F_2,49.79_=2.59, *p*=.085), Sex (F_1,27.11_=1.58, *p*=.219), and Age (F_1,26.27_=2.88, *p*=.102). Before Holm correction, but not after, there was an increased MN-score for night work in warm white light (EMM = 28.3, 95% CI = 24.0–32.6; *p*/*p*^holm^=.030/.090) compared to daytime (EMM = 23.8, 95% CI = 19.4–28.1). The MN-score for blue-enriched white light (EMM = 26.9, 95% CI = 22.6–31.1) did not differ significantly (*p*/*p*^holm^=.131/.263) compared to daytime. The MN-score during night work did not differ significantly (*p*/*p*^holm^=.468/.468) between the light conditions. The final model with Time-of-day (Figure 2(B); right panel) as a fixed factor (Sex and Age not retained) revealed a significant effect of Time-of-day (F_1,50.92_=4.49, *p*=.039, Marginal R^2^/Conditional R^2^=0.024/0.595), with an increased MN-score for night work (EMM = 26.8, 95% CI = 23.1–30.5) compared to daytime (EMM = 23.0, 95% CI = 18.7–27.3).

None of the fixed factors/predictors improved the model fit compared with the random effects model for the PI-score. The model including Condition (F_2,49.94_=0.17, *p*=.846) indicated similar PI-scores (Table 1, Figure 2(C); left panel) for night work in both blue-enriched white light (EMM = 18.6, 95% CI = 15.2–21.9), warm white light (EMM = 18.6, 95% CI = 15.2–22.0), and daytime (EMM = 17.6, 95% CI = 14.1–21.1). All pairwise comparisons had *p*/*p*^holm^≥.611/1.000. The final model with Time-of-day (Figure 2(C); right panel) as a fixed factor showed no significant effect of Time-of-day (F_1,51.14_=0.35, *p*=.559; Marginal R^2^/Conditional R^2^=0.003/0.407), and PI-scores were similar for night work (EMM = 18.6, 95% CI = 15.7–21.4) and daytime (EMM = 17.6, 95% CI = 14.1–21.1).

## Discussion

The present study investigated whether the quality of moral decision-making during simulated night work differed during night work in blue-enriched white light compared to warm white light, and whether moral decision-making differed during night work compared to daytime. The quality of moral decision-making was assessed by measuring the activation of moral schemas on the second consecutive night shift (at 03:30 h), through the evaluation and judgment of five moral dilemmas, using DIT-2. Moral decision-making was assessed during night work in both blue-enriched white light and warm white light, as well as during daytime in a rested state. Among 30 young and healthy adult participants, activation of the post-conventional schema (P-score), that is, higher-level moral reasoning, was significantly reduced during night work in warm white light, but not in blue-enriched white light, compared to daytime. The P-score during night work did not differ significantly between the light conditions. Thus, the participants’ ability to activate the post-conventional schema was reduced during night work, and blue-enriched white light seemed to moderate this reduction to some degree. Activation of the maintaining norms schema (MN-score) during night work did not differ significantly between light conditions, but the MN-score was significantly higher during night work than during daytime. This finding indicates that during night work, participants’ moral decision-making became more rule-oriented than during the daytime. The analyses suggested a higher MN-score during night work in warm white light, but not blue-enriched white light, compared to daytime, but after adjusting for multiple comparisons, the difference no longer remained statistically significant. For activation of the personal interest schema (PI-score), that is, self-oriented thinking, no statistically significant differences were detected.

The present study is the first to investigate whether light conditions during simulated night work affects the quality of moral decision-making. However, previous studies have shown that blue light and/or blue-enriched light during night work can reduce sleepiness and performance impairments evident in other cognitive tests, such as attention and working memory tasks [26–30]. Both attention and working memory are considered requisites for CMD [1], and attention and working memory impairments may also affect moral decision-making abilities. It has been reported previously that the participants in the present study benefitted, yet only to a minor degree, from the blue-enriched white light in terms of reduced performance impairments on a psychomotor vigilance task (sustained attention test) and a digit symbol substitution test (attention test with working memory requirements) [28]. Although the mechanisms are unclear, it is possible that attention and working memory improvements with blue-enriched white light also affect the ability to judge moral dilemmas. There was only an indication of minor effects of light condition on moral decision-making, with blue-enriched white light seen as a possible moderator of the association between night work and reduced activation of post-conventional schema, compared to daytime. Previously, no significant differences were reported in terms of daytime sleep and circadian phase shift (i.e. melatonin rhythm) between the light conditions [28], hence the findings cannot be attributed to differences in circadian rhythms or sleep. Considering the previous findings, and the present finding of minor effects of the light conditions, it is likely that the light conditions were not sufficiently different to yield clear and significant effects. In this study, relatively high light levels (vertical photopic illuminance: ∼200 lx), compliant with European standards for most interior areas [38], were used. It has been suggested that the spectral distribution may be more important for inducing NIF responses at lower light levels, for example, <200 lx [39]. Although melanopic EDI was much higher for blue-enriched white light (174 lx) than for warm white light (78 lx), it has been indicated previously that exposure to <100 lx melanopic EDI can also induce substantial alerting responses [40]. Although it is not clear whether the same applies to moral decision-making, it is possible that stronger effects could have been seen with lower light levels in the present study, as well as with larger differences between light conditions. On the other hand, in the study by Motamedzadeh et al. [27], even higher photopic illuminance (∼350 lx) was used concurrently with higher color temperatures (4000, 6500 and 17 000 K), and improved alertness and cognitive performance were reported, especially for highly blue-enriched (17 000 K) light compared to traditional (4000 K) light. In the present study, the light conditions were photon-matched, with similar photon densities (∼1.6 x 10^14^ photons/cm^2^/s) for both light conditions, while Motamedzadeh et al. [27] used similar photopic illuminance across conditions. Thus, the relative difference in melanopic EDI between the blue-enriched (17 000 K) light and the 4000 K light was probably higher in the study by Motamedzadeh et al. [27], even though the 4000 K light had a higher color temperature than in the present study (2500 K). Sletten et al. [26] reported improved alertness and cognitive performance for blue-enriched light (17 000 K) with relatively low photopic illuminance (106 lx), but with a 4.5-fold increase in melanopic EDI (from 24 lx to 108 lx), compared to the control condition (photopic illuminance: 44 lx, 4000 K). This is quite different from the present study, in which there was *a* ∼2.2-fold increase in melanopic EDI for blue-enriched white light compared to the warm white light. Also, in a study simulating one night shift in narrow-bandwidth short-wavelength light (blue; melanopic EDI: ∼585 lx) and long-wavelength light (red; melanopic EDI: ∼4 lx), with similar photon density (∼2.8 x 10^14^ photons/cm^2^/s), relatively large differences on the PVT were reported [29]. Compared to the present study, the relative differences between the short-wavelength light conditions (e.g. difference in melanopic EDI) were very large in the aforementioned study. Until more studies have investigated whether light interventions during night work affect moral decision-making, evaluation of the effects of light levels on moral decision-making should be tentatively considered.

Previous research on moral decision-making during night work has been restricted. Nevertheless, it is well established that sleep deprivation affects moral decision-making [12–14]. The results in the present study, that is, lower P-scores and higher MN-scores during night work (compared to daytime), follow a pattern similar to that reported for military cadets, where sleep was restricted to 2.5 h per night for five days [14]. However, the relative difference in the P-scores between night work and daytime was considerably smaller in the present study. Note that exposure to the night work schedule in the present study must be considered mild, yet much more relevant for shift workers, compared to the relatively rare and severe partial sleep deprivation in the study by Olsen et al. [14]. Hence, it is not surprising that the difference in P-scores between night work and daytime was relatively small. The participants in the present study had high P-scores (EMM = 51.2, 95% CI = 45.9–56.5) in the daytime (rested) condition, more comparable to the “high P-score” subgroup (average = 51.42, SD = 10.17) than the overall P-score (average = 35.35, SD = 14.12) for the rested condition in Olsen et al. [14]. Olsen et al. [14] reported that those displaying high levels of moral reasoning (i.e. high P-score) while rested, had a larger decrease in the P-score from the rested state to the sleep-deprived state. While Olsen et al. [14] studied mainly male military cadets, the participants in the present study were mainly university students (predominantly females) at the Faculty of Psychology, where a relatively high educational level is required to be enrolled in a study program. Females are known to have higher P-scores than males, and P-scores also increase with education level [18]. The indicated increase in MN-score during night work, that is, more rules-based moral decision-making, suggests that nuanced rules and procedures might be important during night work. Similarly, Olsen et al. [14] noted that incorporating moral standards and concerns into the rules can provide a basis for sound moral judgements also during sleep deprivation.

Although direct comparison is difficult due to different tasks and exposure, the results can be considered to follow in the same vein as in Killgore et al. [12], where two nights (53.5 h) of continuous wakefulness slowed responses to personal moral dilemmas that were emotionally evocative, but not impersonal or non-moral dilemmas. On the other hand, the exposure to two consecutive night shifts in the present study is more comparable to the study by Tempesta et al. [13], where moral reasoning, still using a different task, was assessed after one night (i.e. 26 h) of sleep deprivation. In addition, Tempesta et al. [13] studied mainly female university students, similar to the present study, but not mainly male military personnel, as in Killgore et al. [12] and Olsen et al. [14]. However, the findings in the present study seem to be at odds with those of Tempesta et al. [13], who reported no differences in the response speed for personal moral dilemmas. Still, a notable difference between the present study and the study by Tempesta et al. [13] is testing time. In the present study, moral decision-making was assessed between 03:30–04:00 h during the night shift, while in Tempesta et al. [13], testing was conducted at 10:00 h in the morning. Thus, the circadian factor seems to differ substantially between the present study and the study by Tempesta et al. [13], and it is likely that the circadian factor may have had a greater impact in the present study, as moral decision-making was assessed closer to the expected trough of the participants’ circadian rhythm. Additionally, as moral decision-making was assessed during the second of three consecutive night shifts, participants in the present study were also influenced by the first night shift.

Acute sleep deprivation, sleep loss, and circadian misalignment [5] are mechanisms that might explain the indicated differences in P-scores and MN-scores in the present study. The participants slept on average 7:45 h during the three nights prior to the first night shift. Previously, it was reported that daytime sleep duration after the first night shift was approximately 6 h for both light conditions [28]. Thus, the participants were exposed to acute sleep deprivation (approximately 24 h) in connection with the first night shift, experiencing approximately 1–2 h reduced sleep duration during the day after the first shift, and moral decision-making was assessed at 03:30 h, close to the trough of the participants’ circadian rhythm. The participants’ average recovery sleep duration (7:50 h) was similar to the baseline sleep duration (∼7.45 h). Thus, sleep duration before the daytime assessment of moral decision-making appears to be normal. However, most participant’s melatonin rhythms shifted by approximately 2 h after night work in both light conditions [28], hence it cannot be ruled out that night work and its concomitant circadian phase shift may have affected the daytime assessment. Based on the present data, conclusions regarding the neural mechanisms cannot be inferred. However, previous studies have shown that activity in the PFC region increases when judging moral dilemmas [9,10], and that sleep deprivation reduces activity in PFC regions [11]. As such, it is possible that night work affected participants’ ability to activate PFC regions that are important for moral decision-making.

This study has some limitations that should be considered in future studies. The participants in the present study were young and healthy university students, and were unlikely to be representative of real-life night workers. For instance, the participants showed a relatively high level of mature moral reasoning that may not be similar to that of an average worker [18]. In addition, two-thirds of the sample were female, and more equal gender distributions should be investigated. There were relatively few participants in the present study, indicating that low power may be an issue. Originally, the sample size was determined based on power analyses for measures (e.g. cognitive tests) repeated several times in each night shift and condition, while the present measure (DIT-2) was completed three times (once in each condition). In terms of ecological validity, this was a semi-naturalistic study in which participants came to work in the laboratory before going home to sleep after night shifts. However, the simulated night shifts were not realistic for many real-life workplaces. For instance, healthcare workers during a night shift may face more challenges and be more active than during laboratory trials characterized by sedentary behavior and little activity. On the other hand, the laboratory setting can be comparable to some parts of healthcare workers’ tasks (e.g. surveillance of patients) and other work settings (e.g. control room workers). It must also be noted that although the laboratory was uniformly lit, the precise light exposure for each participant was not measured. The reported light conditions represent the average for the workplaces, and we did not control each participants’ posture and direction of gaze. Another issue with the laboratory setting and use of DIT-2 is that the participants were not facing any real consequences due to decisions about the moral dilemmas. In terms of study design, the light conditions were counterbalanced, ensuring minimal crossover effects, but the daytime completion of DIT-2 took place following the last night work period for all participants. Considering that the participants’ circadian rhythms were shifted by approximately two hours during the night work periods, it would have been more appropriate to conduct the daytime assessment before the night shifts. Moreover, while it is not supposed to be possible to fake a good score on the DIT-2 test, the repeated administration of the test may have had an impact. It has been suggested that the DIT-2 test can serve as both an assessment tool and as an intervention to facilitate the development of moral reasoning [41], although the latter normally requires a longer time span. It is important that future studies, using similar study designs, ensure that the order of conditions is fully counterbalanced to avoid potential biases.

## Conclusion

The present study expands previous findings of sleep deprivation effects on moral reasoning abilities by providing evidence that blue-enriched lighting during night shifts might moderate a negative impact of night work on the quality of moral decision-making. It was also indicated that moral decision-making may decrease on the second night shift in a common night work schedule, which consists of three consecutive night shifts compared to daytime. Although only minor yet statistically significant differences have been reported, the findings are important, as a reduced ability to engage in moral reasoning may have serious consequences in work settings where workers have to make difficult moral decisions (e.g. the health sector). Given the widespread use of night work across several sectors, interventions that can reduce is negative impacts are warranted. This study present the first evidence suggesting that exposure to blue-enriched white light during night work may be beneficial for the quality of moral decision-making. However, several limitations of the present investigation underscore the need for further studies to validate these preliminary findings.

## Supplementary Material

Supplemental Material

## Data Availability

Data from this study are available from the corresponding author upon reasonable request.

## References

[1] Garrigan B, Adlam ALR, Langdon PE. Moral decision-making and moral development: toward an integrative framework. Dev Rev. 2018;49:1–12. doi: 10.1016/j.dr.2018.06.001.

[2] Ganesan S, Magee M, Stone JE, et al. The impact of shift work on sleep, alertness and performance in healthcare workers. Sci Rep. 2019;9(1):4635. doi: 10.1038/s41598-019-40914-x. 30874565 PMC6420632

[3] Åkerstedt T, Wright KP. Jr. Sleep loss and fatigue in shift work and shift work disorder. Sleep Med Clin. 2009;4(2):257–271., doi: 10.1016/j.jsmc.2009.03.001. 20640236 PMC2904525

[4] McHill AW, Wright KP. Jr. Cognitive impairments during the transition to working at night and on subsequent night shifts. J Biol Rhythms. 2019;34(4):432–446. doi: 10.1177/0748730419848552. 31072264 PMC7241942

[5] Satterfield BC, Killgore WDS. Sleep loss, executive function, and decision-making. In: Grandner MA, editor. Sleep and health. London, UK: Academic Press, an imprint of Elsevier; 2019. p. 339–358.

[6] Wickens CD, Hutchins SD, Laux L, et al. The impact of sleep disruption on complex cognitive tasks: a Meta-Analysis. Hum Factors. 2015;57(6):930–946. doi: 10.1177/0018720815571935. 25850114

[7] Harrison Y, Horne JA. The impact of sleep deprivation on decision making: a review. J Exp Psychol Appl. 2000;6(3):236–249. doi: 10.1037/1076-898x.6.3.236. 11014055

[8] Kolk SM, Rakic P. Development of prefrontal cortex. Neuropsychopharmacology. 2022;47(1):41–57. doi: 10.1038/s41386-021-01137-9. 34645980 PMC8511863

[9] Greene JD, Sommerville RB, Nystrom LE, et al. An fMRI investigation of emotional engagement in moral judgment. Science. 2001;293(5537):2105–2108. doi: 10.1126/science.1062872. 11557895

[10] Greene JD, Nystrom LE, Engell AD, et al. The neural bases of cognitive conflict and control in moral judgment. Neuron. 2004;44(2):389–400. doi: 10.1016/j.neuron.2004.09.027. 15473975

[11] Thomas M, Sing H, Belenky G, et al. Neural basis of alertness and cognitive performance impairments during sleepiness. I. Effects of 24 h of sleep deprivation on waking human regional brain activity. J Sleep Res. 2000;9(4):335–352. doi: 10.1046/j.1365-2869.2000.00225.x. 11123521

[12] Killgore WD, Killgore DB, Day LM, et al. The effects of 53 hours of sleep deprivation on moral judgment. Sleep. 2007;30(3):345–352. doi: 10.1093/sleep/30.3.345. 17425231

[13] Tempesta D, Couyoumdjian A, Moroni F, et al. The impact of one night of sleep deprivation on moral judgments. Soc Neurosci. 2012;7(3):292–300. doi: 10.1080/17470919.2011.614002. 21943064

[14] Olsen OK, Pallesen S, Eid J. The impact of partial sleep deprivation on moral reasoning in military officers. Sleep. 2010;33(8):1086–1090. doi: 10.1093/sleep/33.8.1086. 20815191 PMC2910538

[15] Olsen OK, Pallesen S, Espevik R. The impact of partial sleep deprivation on military naval officers’ ability to anticipate moral and tactical problems in a simulated Maritime combat operation. Int Marit Health. 2013;64(2):61–65. 23788221

[16] Barnes CM, Gunia BC, Wagner DT. Sleep and moral awareness. J Sleep Res. 2015;24(2):181–188. doi: 10.1111/jsr.12231. 25159702

[17] Rest JR, Narvaez D, Bebeau MJ, et al. Postconventional moral thinking: a neo-Kohlbergian approach. Mahwah, NJ: Lawrence Erlbaum Press; 1999.

[18] Gungordu N, Nabizadehchianeh G, O’Connor E, et al. Moral reasoning development: norms for defining issue test-2 (DIT2). Ethics & Behavior. 2023. doi: 10.1080/10508422.2023.2206573.

[19] Pallesen S, Bjorvatn B, Magerøy N, et al. Measures to counteract the negative effects of night work. Scand J Work Environ Health. 2010;36(2):109–120. doi: 10.5271/sjweh.2886. 20011984

[20] Vetter C, Pattison PM, Houser K, et al. A review of human physiological responses to light: implications for the development of integrative lighting solutions. Leukos. 2021;18(3):387–414. doi: 10.1080/15502724.2021.1872383.

[21] Lowden A, Kecklund G. Considerations on how to light the night-shift. Lighting Research & Technology. 2021;53(5):437–452. doi: 10.1177/14771535211012251.

[22] Cajochen C. Alerting effects of light. Sleep Med Rev. 2007;11(6):453–464. doi: 10.1016/j.smrv.2007.07.009. 17936041

[23] Mu YM, Huang XD, Zhu S, et al. Alerting effects of light in healthy individuals: a systematic review and meta-analysis. Neural Regen Res. 2022;17(9):1929–1936. doi: 10.4103/1673-5374.335141. 35142669 PMC8848614

[24] Fisk AS, Tam SKE, Brown LA, et al. Light and cognition: roles for circadian rhythms, sleep, and arousal. Front Neurol. 2018;9:56. doi: 10.3389/fneur.2018.00056. 29479335 PMC5811463

[25] Prayag AS, Münch M, Aeschbach D, et al. Light modulation of human clocks, wake, and sleep. Clocks Sleep. 2019;1(1):193–208. doi: 10.3390/clockssleep1010017. 32342043 PMC7185269

[26] Sletten TL, Raman B, Magee M, et al. A blue-enriched, increased intensity light intervention to improve alertness and performance in rotating night shift workers in an operational setting. Nat Sci Sleep. 2021;13:647–657. doi: 10.2147/NSS.S287097. 34079409 PMC8163632

[27] Motamedzadeh M, Golmohammadi R, Kazemi R, et al. The effect of blue-enriched white light on cognitive performances and sleepiness of night-shift workers: a field study. Physiol Behav. 2017;177:208–214. doi: 10.1016/j.physbeh.2017.05.008. 28495465

[28] Sunde E, Pedersen T, Mrdalj J, et al. Blue-enriched white light improves performance but not subjective alertness and circadian adaptation during three consecutive simulated night shifts. Front Psychol. 2020;11:2172. doi: 10.3389/fpsyg.2020.02172. 33013558 PMC7462016

[29] Sunde E, Pedersen T, Mrdalj J, et al. Alerting and circadian effects of short-wavelength vs. long-wavelength narrow-bandwidth light during a simulated night shift. Clocks Sleep. 2020;2(4):502–522. doi: 10.3390/clockssleep2040037. 33255613 PMC7712639

[30] Sletten TL, Ftouni S, Nicholas CL, et al. Randomised controlled trial of the efficacy of a blue-enriched light intervention to improve alertness and performance in night shift workers. Occup Environ Med. 2017;74(11):792–801. doi: 10.1136/oemed-2016-103818. 28630378

[31] CIE. System for metrology of optical radiation for ipRGC-influenced responses to light. International Standard CIE S 026/E:2018. Vienna, Austria: International Commission on Illumination (CIE); 2018.

[32] Rest JR, Narvaez D, Thoma SJ, et al. DIT2: devising and testing a revised instrument of moral judgment. J Educ Psychol. 1999;91(4):644–659. doi: 10.1037/0022-0663.91.4.644.

[33] Olsen OK, Eid J, Johnsen BH. Moral behavior and transformational leadership in norwegian naval cadets. Mil Psychol. 2006;18(sup1):S37–S56. doi: 10.1207/s15327876mp1803s_4.

[34] Bates D, Mächler M, Bolker B, et al. Fitting linear mixed-effects models using lme4. J Stat Soft. 2015;67(1):1–48. doi: 10.18637/jss.v067.i01.

[35] R Core Team. R: a language and environment for statistical computing. R Foundation for Statistical Computing. Vienna, Austria 2022.

[36] Kuznetsova A, Brockhoff PB, Christensen RHB. lmerTest package: tests in linear mixed effects models. J Stat Soft. 2017;82(13):1–26. doi: 10.18637/jss.v082.i13.

[37] Holm S. A simple sequentially rejective multiple test procedure. Scand J Stat. 1979;6(2):65–70.

[38] CEN. Light and lighting - Lighting of work places - Part 1: indoor work places. Brussels: European Commitee for Standardization; 2011.

[39] Cajochen C, Reichert C, Maire M, et al. Evidence that homeostatic sleep regulation depends on ambient lighting conditions during wakefulness. Clocks Sleep. 2019;1(4):517–531. doi: 10.3390/clockssleep1040040. 33089184 PMC7445844

[40] Brown TM. Melanopic illuminance defines the magnitude of human circadian light responses under a wide range of conditions. J Pineal Res. 2020;69(1):e12655. doi: 10.1111/jpi.12655. 32248548

[41] Mayhew MJ, Pascarella ET, Trolian T, et al. Measurements matter: taking the DIT-2 multiple times and college students’ moral reasoning development. Res High Educ. 2015;56(4):378–396. doi: 10.1007/s11162-014-9348-5.

