# Supplementary material for: Moral decision-making at night and the impact of night work with blue-enriched white light or warm white light: a counterbalanced crossover study
Source: Ann Med. 2024 Apr 18;56(1):2331054. doi: 10.1080/07853890.2024.2331054 (PMC11028009; doi:10.1080/07853890.2024.2331054)
Supplement: Supplemental Material [file IANN_A_2331054_SM4691.docx]

**Supplementary material**

**Table S1**. P-score model comparison and building process for the models a) Condition as a fixed effect, and models b) Time-of-day as a fixed effect

| Sampling Units | | N total obs=80  N Subjects=30 | | | | | | | | |
| --- | --- | --- | --- | --- | --- | --- | --- | --- | --- | --- |
|  | | | | | | | | | | |
| **Model specification** | **Model name** | **Nested / simpler model** | **Fixed effects added** | **Random effects** | **Model fit** | | | | **LRT against nested** | |
|  |  |  |  | **Subjects** | **AIC** | **BIC** | **LL** | **N par** | **df** | **X2** |
| RE only | Null | - | - | intercepts | 627.57 | 634.71 | -310.78 | 3 |  |  |
|  | | | | | | | | | | |
| FE main effects | Main effects 1a | Null | condition | intercepts | 622.43 | 634.34 | -306.22 | 5 | 2 | **9.14*** |
| FE main effects | Main effects 1b | Null | time-of-day | intercepts | 622.69 | 632.22 | -307.35 | 4 | 1 | **6.87**** |
|  | | | | | | | | | | |
| Two-way interactions | Interaction effects 1a | Main effects 1a | condition x order | intercepts | 624.25 | 643.31 | -304.12 | 8 | 3 | 4.18 |
| Two-way interactions | Interaction effects 1b | Main effects 1b | time-of-day x order | intercepts | 623.58 | 637.87 | -305.79 | 6 | 2 | 3.12 |
|  | | | | | | | | | | |
| FE main effects | Main effects 2a | Main effects 1a | condition + sex + age | intercepts | 623.71 | 640.39 | -304.86 | 7 | 2 | 2.72 |
| FE main effects | Main effects 2b | Main effects 1b | time-of-day + sex + age | intercepts | 623.95 | 638.24 | -305.97 | 6 | 2 | 2.75 |
|  | | | | | | | | | | |
| Two-way interactions | Interaction effects 2a | Main effects 1a | condition x (sex + age) | intercepts | 630.46 | 656.66 | -304.23 | 11 | 6 | 3.97 |
| Two-way interactions | Interaction effects 2b | Main effects 1b | time-of-day x (sex + age) | intercepts | 627.87 | 646.93 | -305.94 | 8 | 4 | 2.82 |
| Condition: warm white light (night work) vs. blue-enriched white light (night work) vs. daytime (rested). Added as fixed effect in (a) models.  Time-of-day: night work vs. daytime (rested). Added as fixed effect in (b) models (grey).  Order: starting with warm white light vs. starting with blue-enriched white light.  Sex: male vs. female.  Age: continuous centered variable.  *, **: p<.05, p<.01 | | | | | | | | | | |

**Table S2**. MN-score model comparison and building process for the models a) Condition as a fixed effect, and models b) Time-of-day as a fixed effect

| Sampling Units | | N total obs=80  N Subjects=30 | | | | | | | | |
| --- | --- | --- | --- | --- | --- | --- | --- | --- | --- | --- |
|  | | | | | | | | | | |
| **Model specification** | **Model name** | **Nested / simpler model** | **Fixed Effects added** | **Random Effects** | **Model fit** | | | | **LRT against nested** | |
|  |  |  |  | **Subjects** | **AIC** | **BIC** | **LL** | **N par** | **df** | **X2** |
| RE only | Null | - | - | intercepts | 596.56 | 603.70 | -295.28 | 3 |  |  |
|  | | | | | | | | | | |
| FE main effects | Main effects 1a | Null | condition | intercepts | 595.60 | 607.51 | -292.80 | 5 | 2 | 4.96 |
| FE main effects | Main effects 1b | Null | time-of-day | intercepts | 594.14 | 603.66 | -293.07 | 4 | 1 | **4.42*** |
|  | | | | | | | | | | |
| Two-way interactions | Interaction effects 1a | Main effects 1a | condition x order | intercepts | 597.2 | 616.25 | -290.60 | 8 | 3 | 4.40 |
| Two-way interactions | Interaction effects 1b | Main effects 1b | time-of-day x order | intercepts | 594.27 | 608.57 | -291.14 | 6 | 2 | 3.86 |
|  | | | | | | | | | | |
| FE main effects | Main effects 2a | Null | condition + sex + age | intercepts | 594.15 | 610.82 | -290.07 | 7 | 4 | **10.41*** |
| FE main effects | Main effects 2b | Main effects 1b | time-of-day + sex + age | intercepts | 592.67 | 606.96 | -290.33 | 6 | 2 | 5.47 |
|  | | | | | | | | | | |
| Two-way interactions | Interaction effects 2a | Main effects 2a | condition x (sex + age) | intercepts | 599.74 | 625.94 | -288.87 | 11 | 4 | 2.41 |
| Two-way interactions | Interaction effects 2b | Main effects 1b | time-of-day x (sex + age) | intercepts | 594.58 | 613.64 | -289.29 | 8 | 4 | 7.55 |
| Condition: warm white light (night work) vs. blue-enriched white light (night work) vs. daytime (rested). Added as fixed effect in (a) models.  Time-of-day: night work vs. daytime (rested). Added as fixed effect in (b) models (grey).  Order: starting with warm white light vs. starting with blue-enriched white light.  Sex: male vs. female.  Age: continuous centered variable.  *: p<.05 | | | | | | | | | | |

**Table S3**. PI-score model comparison and building process for the models a) Condition as a fixed effect, and models b) Time-of-day as a fixed effect

| Sampling Units | | N total obs=79  N Subjects=30 | | | | | | | | |
| --- | --- | --- | --- | --- | --- | --- | --- | --- | --- | --- |
|  | | | | | | | | | | |
| **Model specification** | **Model name** | **Nested / simpler model** | **Fixed effects added** | **Random effects** | **Model fit** | | | | **LRT against nested** | |
|  |  |  |  | **Subjects** | **AIC** | **BIC** | **LL** | **N par** | **df** | **X2** |
| RE only | Null | - | - | intercepts | 563.63 | 570.74 | -278.82 | 3 |  |  |
|  | | | | | | | | | | |
| FE main effects | Main effects 1a | Null | condition | intercepts | 567.28 | 579.13 | -278.64 | 5 | 2 | 0.35 |
| FE main effects | Main effects 1b | Null | time-of-day | intercepts | 565.28 | 574.76 | -278.64 | 4 | 1 | 0.35 |
|  | | | | | | | | | | |
| Two-way interactions | Interaction effects 1a | Null | condition x order | intercepts | 572.83 | 591.78 | -278.41 | 8 | 5 | 0.81 |
| Two-way interactions | Interaction effects 1b | Null | time-of-day x order | intercepts | 568.93 | 583.15 | -278.46 | 6 | 3 | 0.70 |
|  | | | | | | | | | | |
| FE main effects | Main effects 2a | Null | condition + sex + age | intercepts | 571.17 | 587.76 | -278.59 | 7 | 4 | 0.46 |
| FE main effects | Main effects 2b | Null | time-of-day + sex + age | intercepts | 569.17 | 583.39 | -278.59 | 6 | 3 | 0.46 |
|  | | | | | | | | | | |
| Two-way interactions | Interaction effects 2a | Null | condition x (sex + age) | intercepts | 576.78 | 602.85 | -277.39 | 11 | 8 | 2.85 |
| Two-way interactions | Interaction effects 2b | Null | time-of-day x (sex + age) | intercepts | 571.25 | 590.20 | -277.62 | 8 | 5 | 2.38 |
| Condition: warm white light (night work) vs. blue-enriched white light (night work) vs. daytime (rested). Added as fixed effect in (a) models.  Time-of-day: night work vs. daytime (rested). Added as fixed effect in (b) models (grey).  Order: starting with warm white light vs. starting with blue-enriched white light.  Sex: male vs. female.  Age: continuous centered variable. | | | | | | | | | | |
